# Distinct methylation levels of mature microRNAs in gastrointestinal cancers

**DOI:** 10.1038/s41467-019-11826-1

**Published:** 2019-08-29

**Authors:** Masamitsu Konno, Jun Koseki, Ayumu Asai, Akira Yamagata, Teppei Shimamura, Daisuke Motooka, Daisuke Okuzaki, Koichi Kawamoto, Tsunekazu Mizushima, Hidetoshi Eguchi, Shuji Takiguchi, Taroh Satoh, Koshi Mimori, Takahiro Ochiya, Yuichiro Doki, Ken Ofusa, Masaki Mori, Hideshi Ishii

**Affiliations:** 1 Department of Frontier Science for Cancer and Chemotherapy, Graduate School of Medicine, Osaka University, Suita, Osaka Japan; 2 Department of Cancer Profiling Discovery/ Medical Data Science, Graduate School of Medicine, Osaka University, Suita, Osaka Japan; 3 Prophoenix Division, Food and Life-Science Laboratory, Idea Consultants, Inc., Osaka-city, Osaka, Japan; 4 Division of Systems Biology, Nagoya University Graduate School of Medicine, Nagoya, Aichi Japan; 5 Genome Information Research Center, Research Institute for Microbial Diseases, Osaka University, Osaka, Japan; 6 Department of Gastroenterological Surgery, Graduate School of Medicine, Osaka University, Suita, Osaka Japan; 7 Department of Gastroenterological Surgery, Nagoya City University Graduate School of Medical Sciences and Medical School, Nagoya, Aichi Japan; 8 Department of Surgery, Kyushu University Beppu Hospital, Beppu, Oita Japan; 9 Division of Molecular and Cellular Medicine, National Cancer Center Research Institute, Tokyo, Japan

**Keywords:** Tumour biomarkers, Tumour biomarkers, Cancer epigenetics, Cancer epigenetics

## Abstract

The biological significance of micro (mi)RNAs has traditionally been evaluated according to their RNA expression levels based on the assumption that miRNAs recognize and regulate their targets in an unvarying fashion. Here we show that a fraction of mature miRNAs including miR-17-5p, -21-5p, and -200c-3p and let-7a-5p harbor methyl marks that potentially alter their stability and target recognition. Importantly, methylation of these miRNAs was significantly increased in cancer tissues as compared to paired normal tissues. Furthermore, miR-17-5p methylation level in serum samples distinguished early pancreatic cancer patients from healthy controls with extremely high sensitivity and specificity. These findings provide a basis for diagnostic strategies for early-stage cancer and add a dimension to our understanding of miRNA biology.

## Abstract (web summary)

In cancer it is assumed that microRNAs recognise and regulate their targets uniformly. Here, the authors show that in gastrointestinal cancers methylation of microRNAs may impact their stability, and that levels of microRNA methylation are distinct in pancreatic cancer patients compared to healthy controls with potential diagnostic implications.

## Introduction

Micro (mi)RNAs are broadly conserved small RNA families that are implicated in a wide variety of pathological processes, including cancer initiation and progression^1,2^. Their aberrant expression in cancer tissues and remarkable stability in body fluids makes miRNAs a useful biomarker for cancer diagnosis^3^. However, miRNA research has traditionally employed RNA expression level to evaluate their biological significance^4^, and the possibility of functional heterogeneity has not been extensively investigated. In this study, we demonstrate the value of using miRNA methylation rather than expression level for cancer diagnosis.

Recent evidence suggests that a substantial fraction of mRNAs and non-coding RNAs undergo chemical modification, which is critical for embryonic development and maintenance of physical states^5–8^; in fact, tRNAs—a distinct class of non-coding RNAs—have a variety of RNA modifications that maintain their stability and proper functioning^9,10^. Interestingly, mature tRNAs are 70–90 nucleotides long, which is similar to the length of most miRNA precursors^11^. This led us to hypothesize that mature miRNAs may also undergo RNA modification. Conventional methylation detection methods such as bisulfite sequencing and methylated RNA immunoprecipitation followed by RNA sequencing (RIP-Seq) can only detect pre-established RNA modifications^8,12^. In this study, we used a non-targeted mass spectrometry sequencing technique that enabled unbiased detection of RNA modifications, leading to the identification of novel methylated cytosines and adenines in several mature miRNAs in cancer cell lines, human tissues and serum.

## Results

### RNA methyltransferases were upregulated in gastrointestinal cancer

**Fig. 1:**
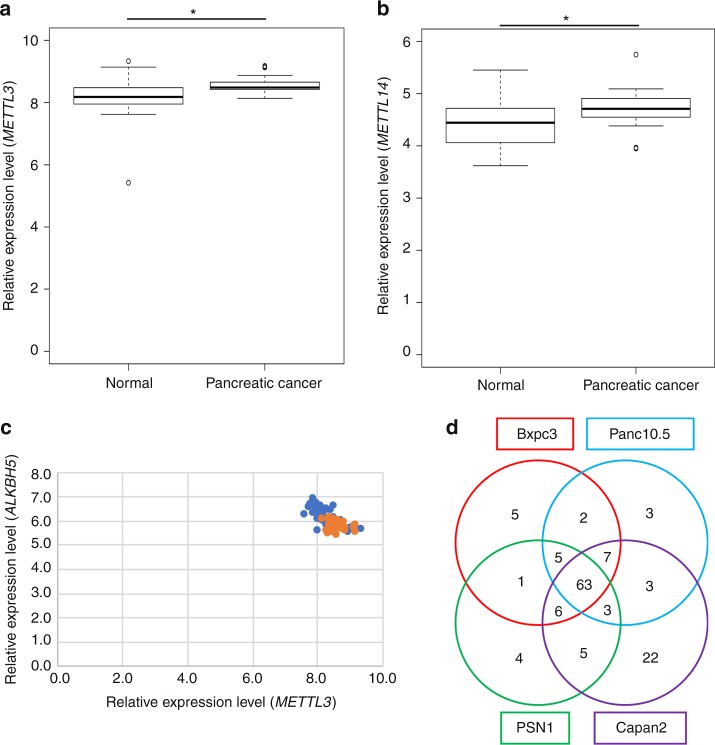
Methylated miRNAs are tended to upregulated in gastrointestinal cancer. **a** Analysis of RNA expression levels of the RNA methylases *METTL3* using a Gene Expression Omnibus (GEO) dataset (GDS4103) derived from pancreatic cancer and paired normal tissue samples from 36 patients. *p= 7.68 × 10^−5^ (Wilcoxon’s *t* test). **b** Analysis of RNA expression levels of the RNA methylase *METTL14* using a Gene Expression Omnibus (GEO) dataset (GDS4103) derived from pancreatic cancer and paired normal tissue samples from 36 patients. *p= 0.007193 (Wilcoxon’s *t* test). **c** Analysis of the correlation between *METTL3* and *ALKBH5* expression levels. Blue and orange points represent normal tissue and Pancreatic cancer tissue, respectively. R value was −0.68 in normal tissue and −0.19 in pancreatic cancer. **d** Methylated miRNA analysis by RIP-Seq using an anti-m6A antibody. The Venn diagram shows that 63 methylated miRNAs were common to four pancreatic cancer cell lines. The box range means from the first quartile to the third quartile. The second quartile means the median of the data. The lower limit of the bar was estimated by “the first quartile − 1.5 × interquartile range”, and the upper limit of the bar was estimated by “the third quartile + 1.5 × interquartile range”

To investigate whether the methylated RNAs are up- or downregulated in cancer cells, we first examined the expression levels of RNA-methylation enzymes; writer proteins such as methyltransferase-like (METTL)3 and METTL14, which are methylation enzymes for adenine^13–15^ and NOP2/Sun RNA methyltransferase family member (NSUN)2, which is a methylation enzyme for cytosine^16^. Although the expression levels were similar between gastrointestinal cancer and normal tissues, the data showed different distribution between gastrointestinal cancer and normal tissues. The expression levels tended to be upregulated in gastrointestinal cancer. (Fig. 1a, b and Supplementary Figs. 1 and 2). Moreover, the expression levels of METTL3 and the adenine demethylation enzyme AlkB homolog (ALKBH)5 were negatively correlated (Fig. 1c), suggesting that RNA methylation tended to increase in gastrointestinal cancer. To quantify the methylated miRNA in gastrointestinal cancer cells, we carried out liquid chromatography-mass spectrometry (MS) analysis^17^ of small RNA fractions of three different cell lines that were size-fractionated from total RNA by ultrafiltration. RNA methyl marks including 5-methylcytosine (5mC), N6-methyladenosine (m6A), 3-methylcytosine, and N1-methyladenine (m1A) were detected in 1–8% of total adenines and cytosines analyzed (See Supplementary Fig. 3). Importantly, the fraction of methylated miRNAs increased upon stimulation with epidermal growth factor in all cell lines examined, suggesting the existence of a systemic regulatory mechanism for RNA modification.

### Methylated microRNAs have altered target inhibitory effects

To clarify the biological significance of mature miRNA methylation, we synthesized miR-200c oligonucleotides with m6A or m5C modifications at all adenines and cytosines, respectively. These oligonucleotides with or without methylation were transfected into the DICER exon 5-disrupted colorectal cancer cell line HCT116 (HCT116^EX5^), which has very low expression levels of endogenous miRNAs^18^. Gene expression profiling revealed that m6A-modified miR-200c-3p did not reduce target mRNA expression level as compared to m5C-modified or non-methylated miR-200c-3p (See Supplementary Fig. 4).

### New-method for detect the methylated microRNAs using MALDI-TOF-MS

**Fig. 2:**
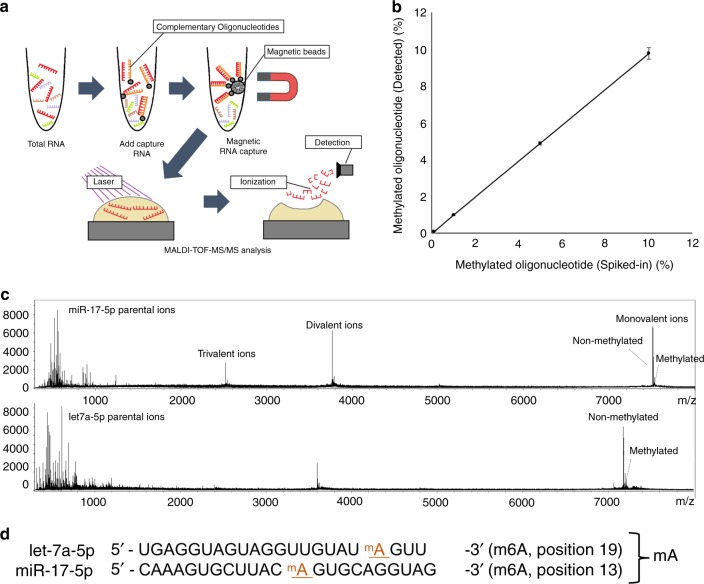
Detection of methylated bases in mature miRNAs. **a** Schematic depiction of the procedure for detecting RNA modifications in mature miRNA sequences. Total small RNA extracted from cells was hybridized with oligonucleotides complementary to target miRNAs on magnetic beads. Captured miRNAs were eluted and applied to sample plates and then analyzed by matrix-assisted laser desorption/ionization time-of-flight tandem MS (MALDI-TOF-MS/MS). **b** Dynamic range of methylated miRNA detection. Synthetic miR-200c-3p oligonucleotides with or without methylation were mixed at the indicated concentrations and analyzed by MALDI-TOF-MS/MS. **c** Mass spectrum of miR-17-5p and let-7a-5p obtained from pancreatic cancer patient-derived tissue. The spectrum shows monovalent, divalent, and trivalent methylated miR-17-5p RNA peaks and monovalent and divalent let-7a-5p RNA peaks (see Methods for details). **d** Position of methylated nucleoside in each miRNA

**Fig. 3:**
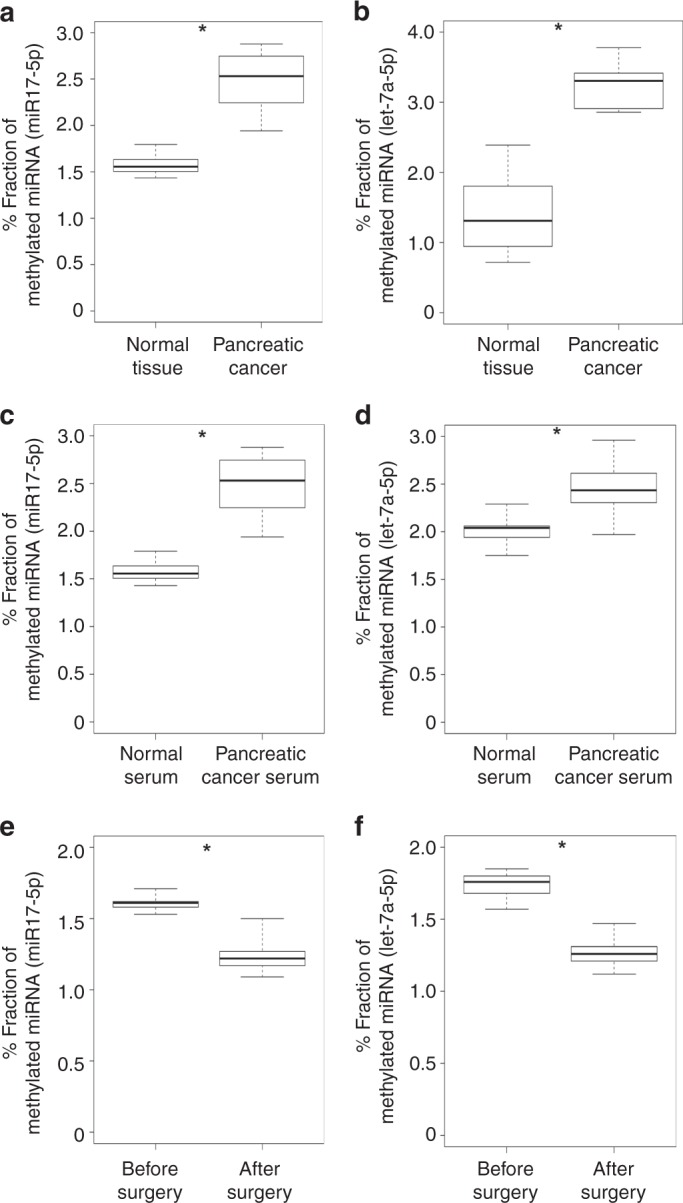
Increased miRNA methylation levels in pancreatic cancer tissue and serum. **a**, **b** Enhancement of miR-17-5p (**a**) and let-7a-5p (**b**) methylation in pancreatic cancer tissue (*n* = 12) relative to paired healthy tissue (*n* = 12). *p < 0.01 (t test). **c**, **d** Fraction of methylated miRNA at a specific position of miR-17-5p (**c**) and let-7a-5p (**d**) in serum derived from pancreatic cancer patients (*n* = 5) and healthy controls (*n* = 5). Healthy control serum was obtained from liver transplantation donors who were confirmed as having no cancer by endoscopy, computed tomography, and by detection of several tumor markers.*p < 0.01 (*t* test). **e**, **f** Fraction of methylated miRNA at a specific position of miR-17-5p (**e**) and let-7a-5p (**f**) in serum derived from pancreatic cancer patients before (*n* = 21) and after (*n* = 21) surgery. Healthy control serum was obtained from liver transplantation donors who were confirmed as having no cancer by endoscopy, computed tomography, and by detection of several tumor markers.*p < 0.01 (*t* test). The box range means from the first quartile to the third quartile. The second quartile means the median of the data. The lower limit of the bar was estimated by “the first quartile − 1.5 × interquartile range”, and the upper limit of the bar was estimated by “the third quartile + 1.5 × interquartile range

Based on our observation that RNA methyltransferases were increased in gastrointestinal cancer cells, we speculated that methylated miRNAs could serve as biomarkers for gastrointestinal cancer. To be find the commonly methylated miRNAs in gastrointestinal cancer, we tried to m6A RIP-Seq analysis of four pancreatic cancer cell lines and identified 63 commonly-methylated miRNAs (Fig. 1d and See Supplementary Table 1). Since conventional RNA-Seq with a next-generation sequencer cannot detect methylated bases in miRNAs, we purified target miRNAs using magnetic beads with bound complementary oligonucleotides and analyzed these by matrix-assisted laser desorption/ionization time-of-flight tandem MS^19–23^ (Fig. 2a, b). Methylated nucleotides were detected as a peak a +14 Da from predicted non-methylated peaks in the mass spectrum; the methylation site was further confirmed by derivatization of nucleotides (see Methods for details). The methylation level of each miRNA was evaluated using synthetic non-methylated (let-7a-5p and miR-17-5p) and methylated (let-7a-5p and miR-17-5p) miRNAs as the ratio between peak intensities of methylated and non-methylated nucleotides. This approach provided a highly sensitive and quantitative measurement of non-methylated and methylated miRNA oligonucleotides (Fig. 2b, See Supplementary Fig. 5 and Supplementary Table 2). We used this method to assess the methylation levels of miRNAs identified by RIP-Seq (Fig. 1d, See Supplementary Table 1) in pancreatic cancer tissue. Let-7a-5p and miR-17-5p had m6A whereas miR-200c-3p and miR-21-3p had 5mC modifications at specific positions in the mature sequence (Fig. 2c, d, See Supplementary Fig. 6). We next measured the methylation levels of these miRNAs in pancreatic and colorectal cancer tissues and paired normal samples and found that methylation was increased in all examined cases whereas no differences in miRNA expression level were detected by quantitative reverse transcription PCR (Fig. 3a, b, See Supplementary Figs 7–10, and Supplementary Tables 3–4). Moreover, the methylation levels of these miRNAs were higher in serum samples from pancreatic and colorectal cancer patients than in those from normal subjects (Fig. 3c, d, See Supplementary Figs. 11–14), and were lower in post- as compared to pre-surgery samples (Fig. 3e, f).

### Predicting structural changes of RISC complex by methylated microRNA

To clarify the biological significance of mature miRNA methylation, we carried out molecular simulations to examine the binding between Argonaute (AGO) protein and miR-17-5p and -200c-3p and let-7a-5p (with or without methylation) as well as structural changes in the complexes. In miR-200c-3p, the 5mC modification at position 9 was close to RNA recognition bases. Although there was no obvious difference between the first six nucleotides of methylated and non-methylated miR-200c-3p in the AGO complex, variations in the binding interaction between AGO and each miRNA were observed around the methyl groups, which enhanced van der Waals interactions with the protein and thereby diminished the surrounding space. At the same time, methyl groups of the cytosine at position 9 disrupted hydrogen bonding with Ser220 of AGO likely through steric hindrance, leading to a positional shift of the guanine at position 8 that was also caused by interaction with Arg761 of AGO (See Supplementary Fig. 15). In miR-17-5p and let-7a-5p, methylated adenines were located away from the RNA-binding site; however, m6A modification causes a large structural change in the whole complex, including around the RNA recognition site, which affects the target RNA recognition efficiency (See Supplementary Figs. 16 and 17). These findings indicate that m6A modification reduces the ability of miRNAs to suppress target mRNA translation.

### Methylated microRNAs become biomarkers of gastrointestinal cancer

**Fig. 4:**
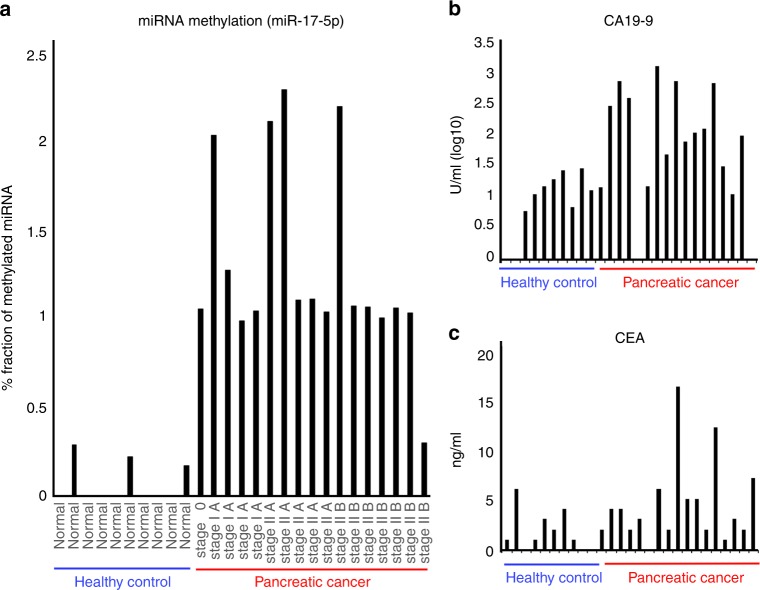
Diagnostic value of methylated miRNA in early-stage pancreatic cancer. **a** Left, bar graph showing the fraction of methylated miRNA of miR-17-5p in serum from pancreatic cancer patients (*n* = 17) and healthy controls (*n* = 10). The clinical stages of pancreatic cancer are indicated at the bottom. Expression below the detection limit is shown as zero. Right, quantitative analysis of carbohydrate antigen (CA19-9) (**b**) and carcinoembryonic antigen (CEA) (**c**) expression in the same set of samples. See Supplementary Table 5 for detailed clinicopathological information of each patient

To evaluate the potential of miRNA methylation as a biomarker for early cancer diagnosis, we examined miRNA methylation levels in serum samples from pancreatic cancer patients and healthy controls. Methylated miR-17-5p was detected in all pancreatic cancer patient samples but was either absent or present only at a low level in controls (Fig. 4a). Moreover, miRNA methylation showed better performance in detecting early-stage pancreatic cancer than established biomarkers such as carbohydrate antigen 19-9 (CA19-9) and carcinoembryonic antigen (CEA)^24^ (Fig. 4b, c, See Supplementary Fig. 18, and Supplementary Table 5). Thus, evaluating miRNA methylation and not simply the expression level is a promising diagnostic strategy. To evaluate the methylation of miRNAs as a biomarker, we agree that a relatively large scale study with cancer patients and healthy controls would be necessary for the clinical use. Although the results of this study provide evidence for the biological significance of RNA methylation status in gastrointestinal cancer. Although high-throughput nucleic acid sequencing is currently the gold standard for transcriptome-level analyses, MS enables high-resolution profiling of these chemical modifications, which can aid in the early diagnosis and treatment of cancer. Moreover, elucidating the mechanisms by which methylation regulates miRNA function in the initiation and progression of cancer can lead to the development targeted therapies that can improve patient outcome.

## Methods

### Cell lines and culture

BxPC3, Panc10.5, PSN1, and Capan2 human pancreatic cancer cell lines were obtained from American Type Culture Collection (Manassas, VA, USA). HCT116EX5 cells were a gift from Bert Vogelstein (Johns Hopkins University, Baltimore, MS, USA). Mycoplasma testing was performed using the MycoAlert Mycoplasma Detection Kit (Lonza, Allendale, NJ). Mycoplasma testing confirmed negative results. Cells were cultured in Roswell Park Memorial Institute-1640 medium supplemented with 10% fetal bovine serum (FBS) (Thermo Fisher Scientific, Waltham, MA, USA) at 37 °C in a humidified incubator of 5% CO_2_.

### miRNA immunoprecipitation. miRNA immunoprecipitation

Total RNA was extracted from cells and tissues using TRIzol reagent (Thermo Fisher Scientific) according to the manufacturer’s instructions. Small RNA fraction (<100 nt) including miRNA were separated from total RNA using High Pure miRNA Isolation Kit (Roche, Basel, Switzerland) according to the manufacturer’s instructions. Small RNA immunoprecipitation was performed using anti-m6A antibody (200 microμg/ml) (cat. 202 003, Synaptic Systems, Goettingen, Germany) at 4°C for 2 h. The miRNA-anti-m6A-antibody complex was incubated with Dynabeads Protein G (Thermo Fisher Scientific) at 4 °C for 2 h. The mixture was obtained by magnetic separation. M6A containing microRNA was eluted from the mixture using 6.7 mM N6-Methyladenosine 5-monophosphate sodium salt (Sigma-aldrich, St. Louis, MO, USA) at 4 °C for 2 h.

### RNA microarray

After assessment for quality, 500 ng of the extracted totalRNA was labeled with Cyanine-3 (Cy3) using the Low Input Quick Amp Labeling Kit (Agilent Technologies, Santa Clara, CA, USA). Dye incorporation and cRNA yield were assessed using the NanoDrop ND-2000 Spectrophotometer. The labeled RNAs were hybridized onto Human Oligo chip 25k (TORAY, Tokyo, Japan) for 17 h at 65 °C in a rotating. After hybridization, microarrays were stringently washed for 1 min at room temperature with GE Wash Buffer 1 (Agilent Technologies) followed by GE Wash buffer 2 for 1 min at 37 °C (Agilent Technologies) and then immediately dried by brief centrifugation. The fluorescent signals were then scanned with the Agilent DNA Microarray Scanner. The data have been submitted to the Gene Expression Omnibus database of National Center for Biotechnology Information (accession no. GSE134794).

### miRNA sequencing

miRNA libraries were constructed following manufacturer instructions using the NEBNext Multiplex Small RNA Library Prep Set for Illumina (New England Biolabs, Ipswich, MA, USA) and sequenced by the HiSeq 2500 platform (Illumina, San Diego, CA, USA). After removing 3′ adapters with the FASTX-toolkit, reads were aligned to the mouse and human reference genomes and transcriptomes using TopHat2 (https://ccb.jhu.edu/software/tophat/index.shtml). The human (hg38) reference sequences used for this purpose were downloaded from the University of California at Santa Cruz genome browser. We calculated the reads per kilobase of exon per million reads mapped reads using Cufflinks (http://cole-trapnell-lab.github.io/cufflinks/). Raw reads from these samples have been submitted to the Gene Expression Omnibus database of National Center for Biotechnology Information (accession no. GSE119014).

### miRNA capture

Total RNA was extracted from cells and tissues using TRIzol reagent (Thermo Fisher Scientific) according to the manufacturer’s instructions and annealed to single-strand DNA oligonucleotides complementary to target miRNAs that were adenine-methylated at the 5′ end via a C6 linker. The mixture was heated to 95 °C, then gradually cooled to 30 °C. The miRNA-DNA complex was incubated with Dynabeads M-270 Amine (Thermo Fisher Scientific) at 4 °C for 1 h. The mixture was heat-eluted and the supernatant was obtained by magnetic separation. Lyophilized samples were used for subsequent experiments.

### Matrix-assisted laser desorption/ionization time-of-flight mass spectrometry (MALDI-TOF-MS)

Captured miRNAs were purified with a Zip Tip C18 column (Millipore, Billerica, MA, USA) according to the manufacturer’s protocol. Purified samples were mixed with an aqueous solution of 3-hydroxypicolinic acid (Bruker Daltonics, Bremen, Germany) at a ratio of 1:1 (v/v) and applied to the target plate. One microliter of the mixture was applied to an MTP AnchorChip 384 target plate (Bruker Daltonics) and air-dried at room temperature. MALDI-TOF-MS analysis was performed with an ultrafleXtreme MALDI-TOF/TOF mass spectrometer (Bruker Daltonics) operated in negative ion and reflectron modes. Spectra were manually acquired using FlexControl software (v.3.3.108.0) (Bruker Daltonics). An analysis of the RNAs following hydrazine treatment revealed that the peaks contained m5C^25^. Methylated adenines were determined to be 6 mA by sequential MS analysis following treatment with dimethylsulfate, which preferentially alkylates the N1 of adenines in RNA^26^.

### Epidermal growth factor (EGF) stimulation

Cells were seeded in 6-well plates (2 × 105 cells/well) and incubated with medium containing 10% FBS. After 48 h, the medium was replaced with one containing 1% FBS. After another 24 h, recombinant EGF (Sigma-Aldrich) was added at a concentration of 100 ng/ml; untreated cells served as the control group. The cells were collected 12 h later for analysis.

### Liquid chromatography-tandem MS (LC-MS/MS)

Total RNA was purified by ultrafiltration using an Eclipse XDB-C18 high-performance LC column (Agilent Technologies) with a molecular weight cut-off of 3 kDa. Isolated small RNA fractions were hydrolyzed into nucleosides in a reaction mixture containing benzonase, phosphodiestrase I, and alkaline phosphatase. LC-MS/MS analysis was performed on an Agilent 6400 mass spectrometer (Agilent Technologies).

### Transfection of synthetic oligonucleotides

Double-stranded RNA oligonucleotides with or without methyl groups were synthesized by Gene Design (Osaka, Japan). The sequence of non-methylated and methylated nucleosides was verified by MALDI-TOF-MS/MS. miRNA sequences were obtained from miRBASE (release 21; http://www.mirbase.org/). The sequence of each oligonucleotide is shown in Fig. 2d. Synthetic miRNAs were transfected into cells using Lipofectamine 3000 (Invitrogen) according to the manufacturer’s protocol.

### Expression data analysis

In order to compare the difference of gene expression changes adopted miR-200c-3p of cytosine-methylated and non-methylated forms to, Gene set enrichment analysis (GSEA) was performed using the fgsea package^27^ on R version 3.5.225^28^. In these analyses, we used the original gene set for targets of above micro RNAs based on Tarbase (http://diana.imis.athena-innovation.gr/DianaTools/index.php?r=tarbase/index)^29^, which was decided based on some experimental facts. GSEA was performed on the ranked list for changes between two experimental conditions (non-methylated or methylated micro RNA and control) on gene sets of these target genes.

### Real-time quantitative reverse transcription polymerase chain reaction (qRT-PCR)

The TaqMan MicroRNA Reverse Transcription kit (Applied Biosystems, Foster City, CA, USA) was used for miRNA quantification according to the manufacturer’s protocols.

### Molecular dynamics simulations

To predict the binding of methylated and non-methylated miRNAs to the human AGO2 protein (See Supplementary Figs. 15–17), we used the X-ray structure of AGO2/RNA as the guide complex (PDB ID: 4OLB^30^ and 4W5N^31^). Initially, the RNA-binding bases were substituted with the corresponding bases in each miRNA. For each miRNA complex structure, molecular dynamics simulations were performed under organic conditions (1 atm and ~37 °C). After thermodynamic sampling, energy minimizations were carried out to predict the docking of the structures. All calculations were performed with AMBER99 force field^32^ using the Amber 12 program (http://ambermd.org/)^33^.

### Clinical samples

Colorectal and gastric cancer and paired normal tissue samples (located >5 cm from the malignant region) were obtained during surgery. All patients underwent resection of the primary tumor at Osaka University Hospital (Osaka, Japan) and affiliated hospitals. Blood samples from pancreatic cancer patients were collected before surgery. Healthy controls had no visible lesions by diagnostic imaging and no abnormal findings in the blood examination. Informed consent was obtained from all participants. We have complied with all relevant ethical regulations for work with human participants. The study protocols were approved by the ethics committee of Osaka University (approval nos. 663, 15222-2, and 08226-5).

### Reporting summary

Further information on research design is available in the Nature Research Reporting Summary linked to this article.

## Supplementary information

Supplementary Information

Reporting Summary

## Data Availability

All the data supporting the findings of this study are available within the article and its supplementary information files and from the corresponding author upon reasonable request. Sequence data for Fig. 1d have been deposited in the NCBI GEO data set with accession code GSE119014; Microarray data for Supplementary Fig. 4 have been deposited in the NCBI GEO data set with accession code GSE134794. Public datasets used in this study can be found in GEO under the accession numbers: GDS4103 (https://www.ncbi.nlm.nih.gov/geo/query/acc.cgi?acc=GSE15471), and GDS4382 (https://www.ncbi.nlm.nih.gov/geo/query/acc.cgi?acc=GSE32323). X-ray structure used in this study can be found in PDB ID: 4OLB and 4W5N2 (http://www.rcsb.org/structure/4W5N).
