# Supplementary material for: Distinct methylation levels of mature microRNAs in gastrointestinal cancers
Source: Nat Commun. 2019 Aug 29;10:3888. doi: 10.1038/s41467-019-11826-1 (PMC6715669; doi:10.1038/s41467-019-11826-1)
Supplement: Supplementary file 1 — Supplementary Information [file 41467_2019_11826_MOESM1_ESM.pdf]

Distinct Methylation Levels of Mature MicroRNAs in Gastrointestinal Cancers

Konno et al.

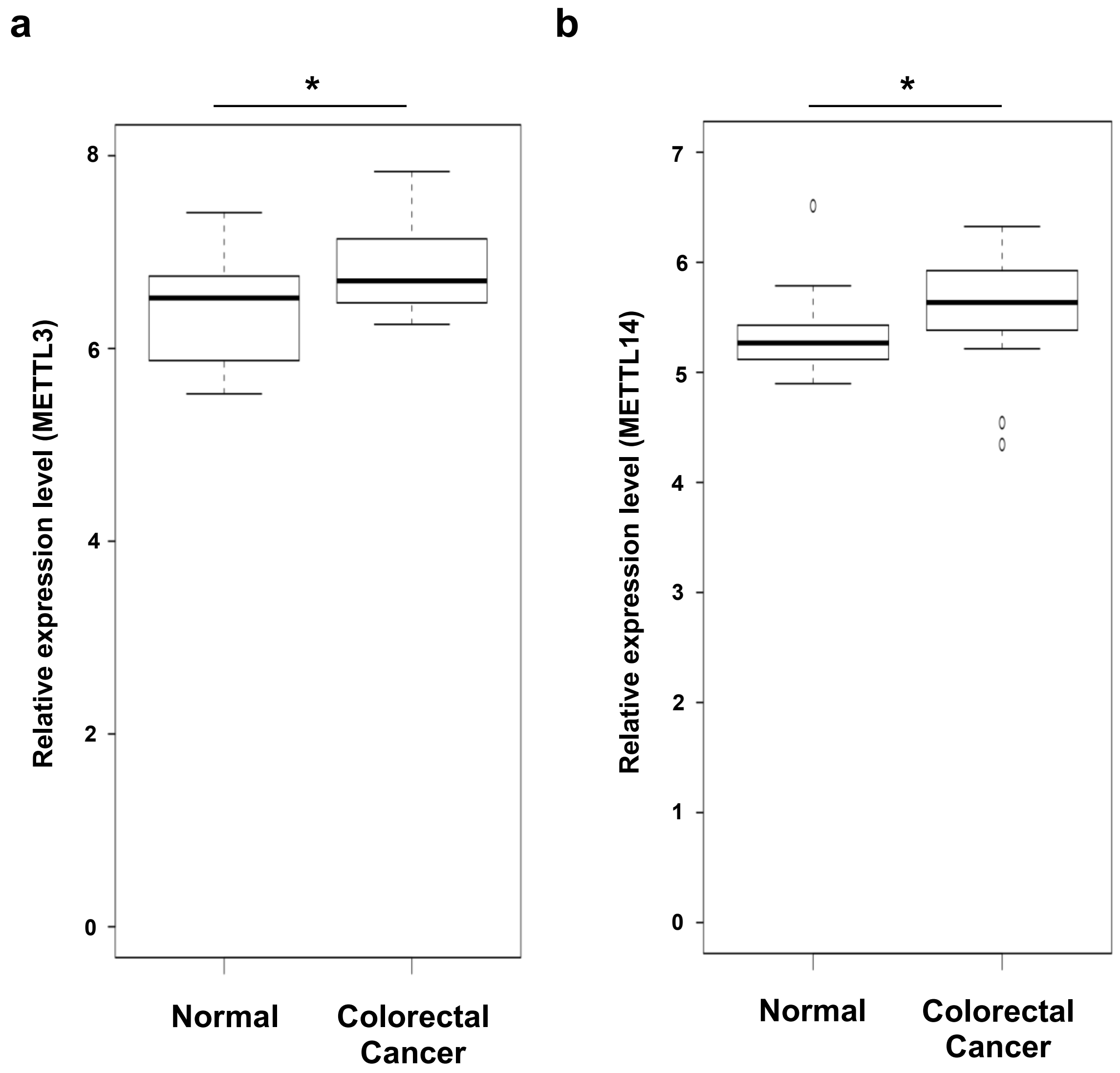

Supplementary Figure 1. (a) Analysis of RNA expression levels of the RNA methylase *METTL3* using a Gene Expression Omnibus (GEO) dataset (GDS4382) derived from colorectal cancer and paired normal tissue samples from 17 patients. *P = 0.06749 (Wilcoxon's t test). (b) Analysis of RNA expression levels of the RNA methylase *METTL14* using a Gene Expression Omnibus (GEO) dataset (GDS4382) derived from colorectal cancer and paired normal tissue samples from 17 patients. *P = 0.01959 (Wilcoxon's t test). The box range means from the first quartile to the third quartile. The second quartile means the median of the data. The lower limit of the bar was estimated by "the first quartile - 1.5 x interquartile range", and the upper limit of the bar was estimated by "the third quartile + 1.5 x interquartile range".

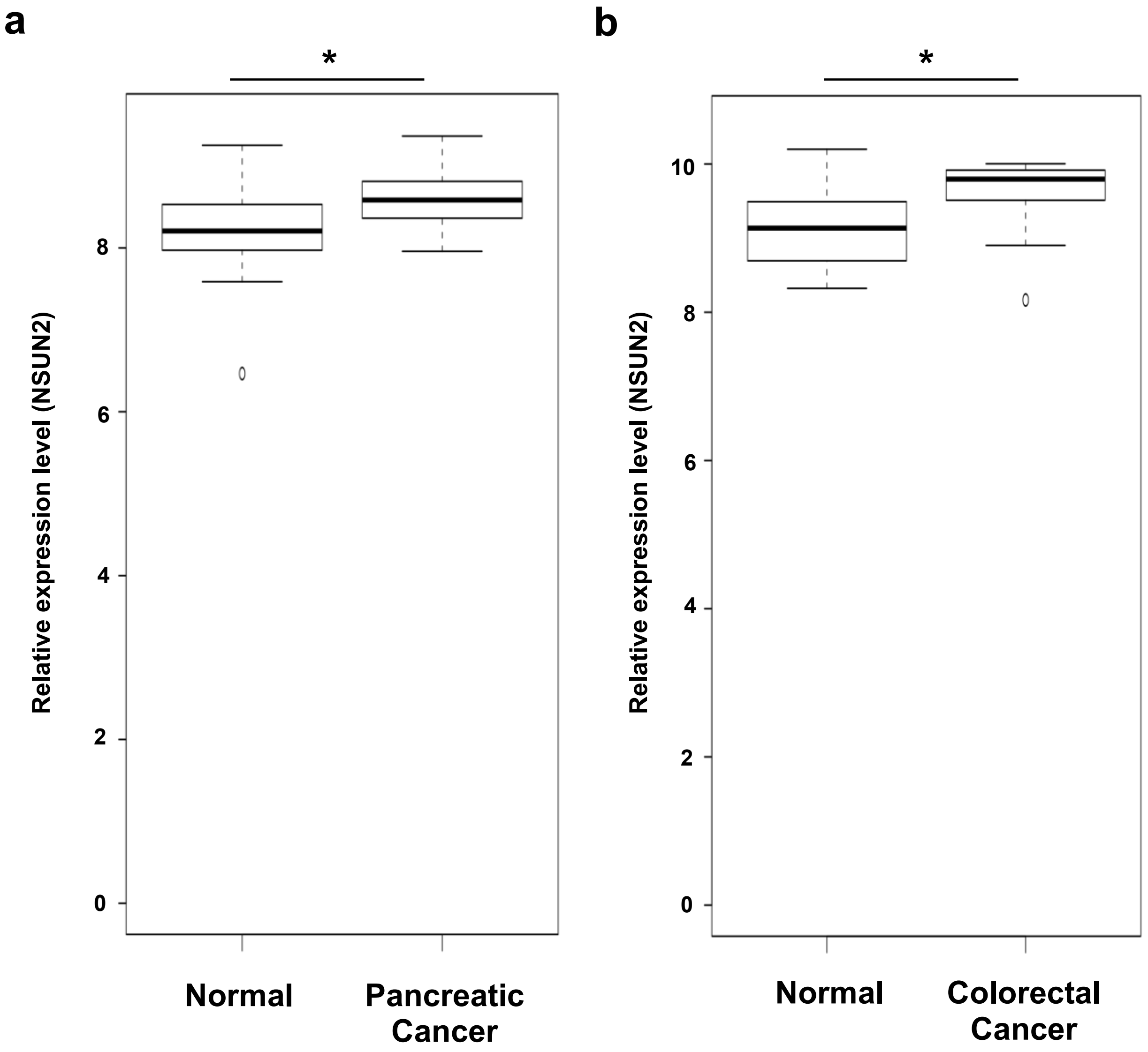

Supplementary Figure 2. (a) Analysis of RNA expression levels of the RNA methylase *NSUN2* using a Gene Expression Omnibus (GEO) dataset (GDS4103) derived from pancreatic cancer and paired normal tissue samples from 36 patients. *P = 1.474×10^{-5} (Wilcoxon's t test). (b) Analysis of RNA expression levels of the RNA methylase *NSUN2* using a Gene Expression Omnibus (GEO) dataset (GDS4382) derived from colorectal cancer and paired normal tissue samples from 17 patients. *P = 0.005394 (Wilcoxon's t test). The box range means from the first quartile to the third quartile. The second quartile means the median of the data. The lower limit of the bar was estimated by "the first quartile - 1.5 x interquartile range", and the upper limit of the bar was estimated by "the third quartile + 1.5 x interquartile range".

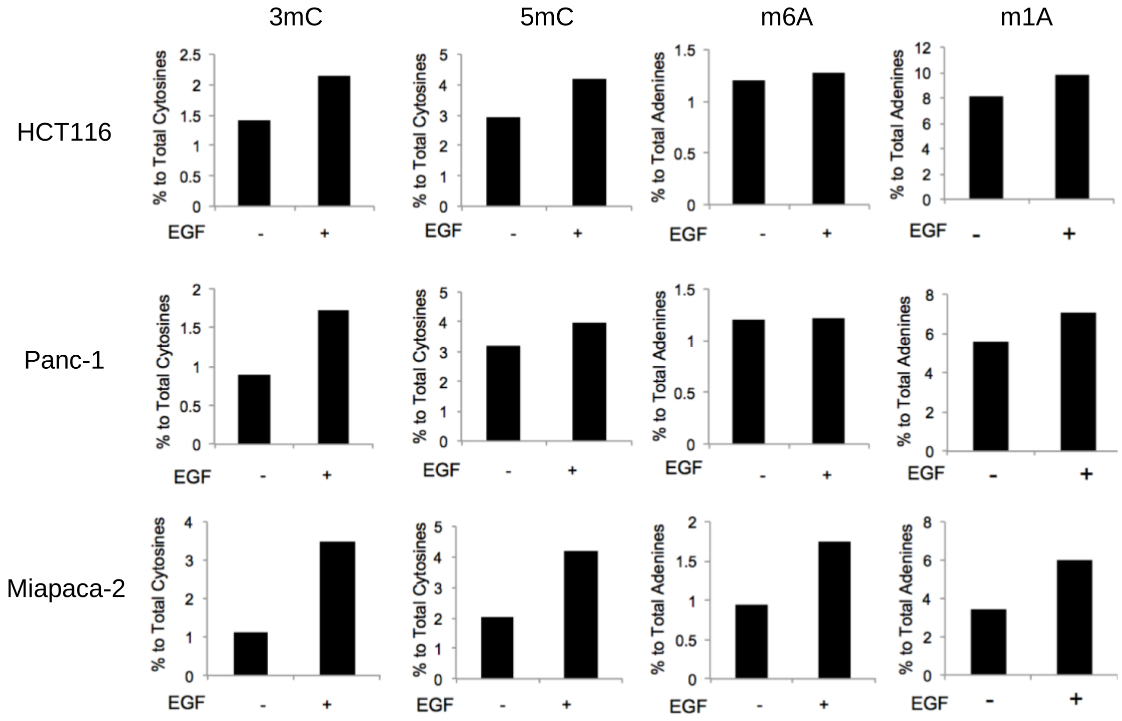

Supplementary Figure 3. Abundance ratios of modified oligonucleotides before and after exposure to EGF. Small RNAs were size-fractionated from total RNA by ultrafiltration in the indicated cell lines, and quantified by liquid chromatography–tandem mass spectrometry. Bar graphs show the ratio of modified to total cytosines and adenines. 3mC, 3-methylcytosine; 5mC, 5-methylcytosine; m6A, N6-methyladenine; m1A, 1-methyladenine.

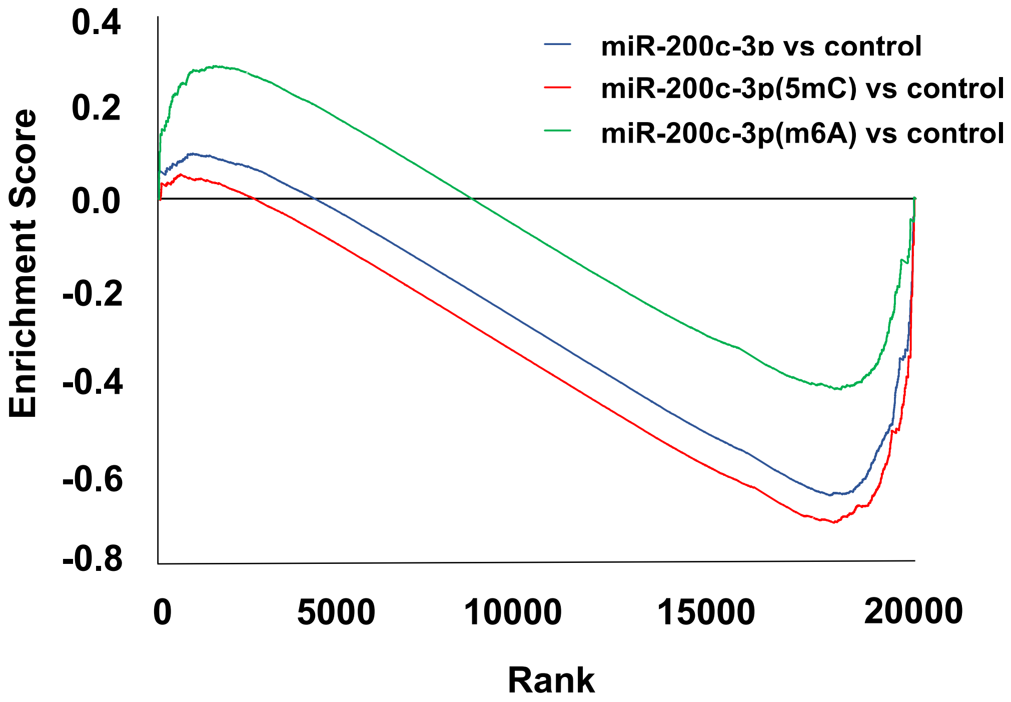

Supplementary Figure 4. Differences in gene suppressor effects of non-methylated miR-200c (blue), miR-200c-3p with 5mC (red), and miR-200c-3p with m6A (green), as determined by gene enrichment analyses. Non-methylated and m5C-modified miR-200c-3p showed potent gene-suppressor effects ($P < 0.005$ and 0.001, respectively; Fisher's exact test), while m6A-modified miR-200c-3p did not suppress target gene expression.

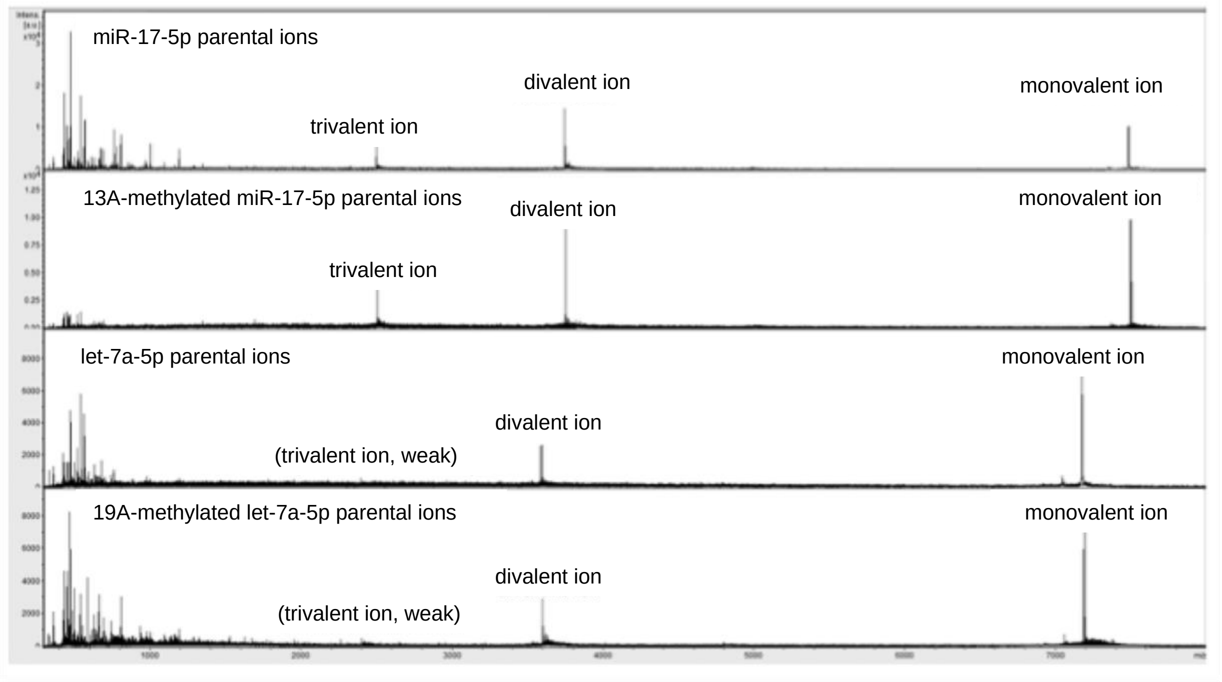

Supplementary Figure 5. Mass spectra obtained for synthesized non-methylated miRNAs (miR-17-5p and let-7a-5p) and methylated miRNAs (13A-methylated miR-17-5p and 19A-methylated let-7a-5p) by MALDI-TOF-MS and MALDI-TOF-MS/MS (let-7a-5p). Five pmoles synthesized RNAs were set on target plate. The spectra show monovalent, divalent, and trivalent ions from methylated and non-methylated miRNAs.

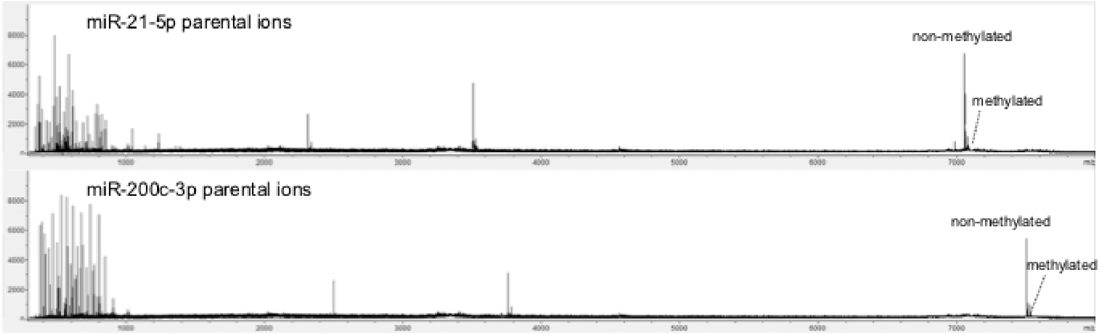

Supplementary Figure 6. Mass spectra obtained from patient-derived pancreatic cancer tissue by MALDI-TOF-MS and MALDI-TOF-MS/MS. The spectra show monovalent, divalent, and trivalent ions from non-methylated and methylated miRNAs.

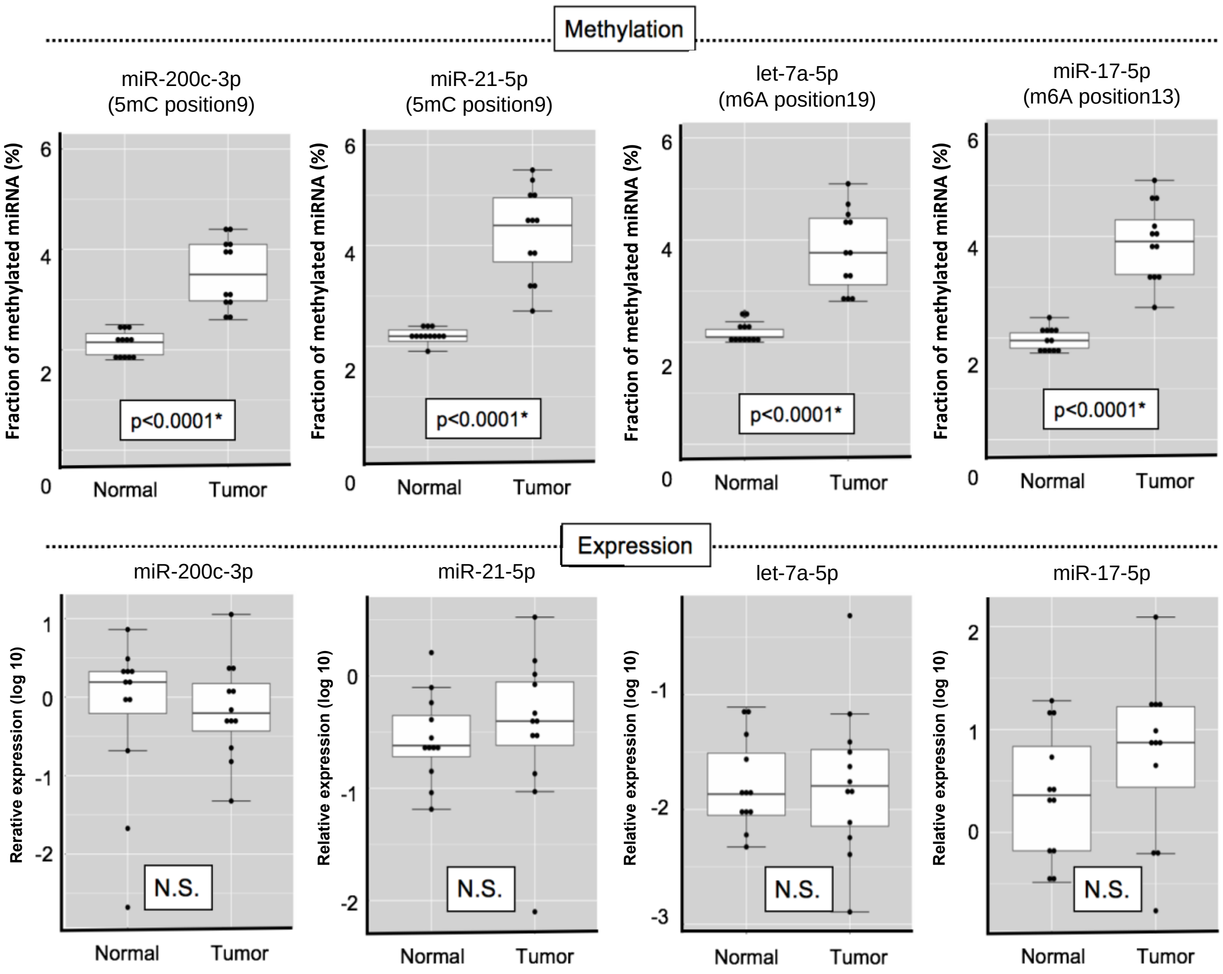

Supplementary Figure 7. (Upper panels) Fraction of methylated miRNAs at specific positions in each miRNA in colorectal cancer and paired normal tissues (n = 12) (related to Supplementary Figure 8). (Lower panels) Expression level of each miRNA in the same samples determined by qRT-PCR and normalized to that of *RNU48* (internal control). Detailed clinicopathological information on each patient can be found in Supplementary Table S3. *P < 0.05 (t test). The box range means from the first quartile to the third quartile. The second quartile means the median of the data. The lower limit of the bar was estimated by "the first quartile - 1.5 x interquartile range", and the upper limit of the bar was estimated by "the third quartile + 1.5 x interquartile range". Points means individual data value.

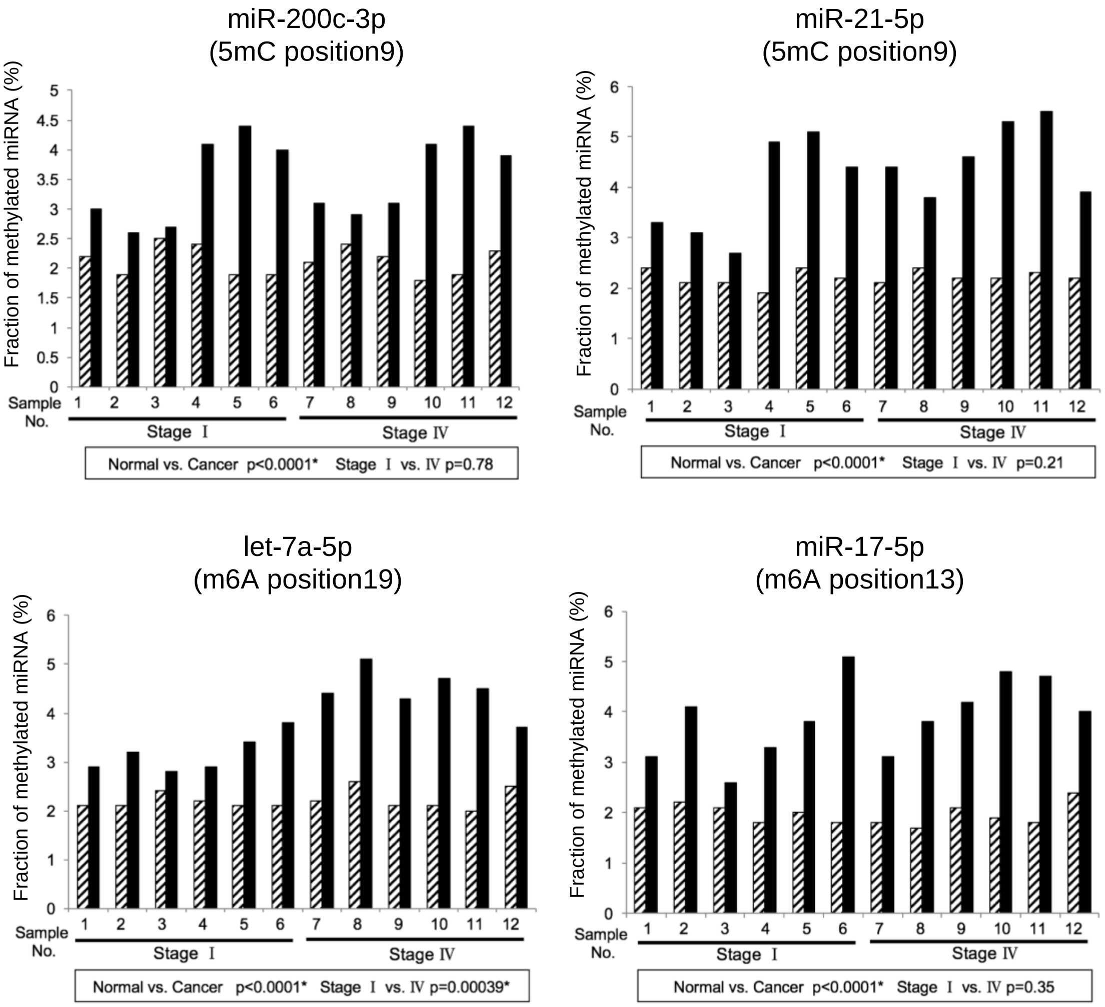

Supplementary Figure 8. Fraction of methylated miRNAs at specific positions in each miRNA in colorectal cancer and paired normal tissues. Numbers below the bar graphs indicate different patients. Solid and hatched bars represent cancer and normal issues, respectively. Samples 1–6 are from stage I and samples 7–12 are from stage IV patients. Detailed clinicopathological information on each patient can be found in Supplementary Table S3. *P < 0.05 (t test).

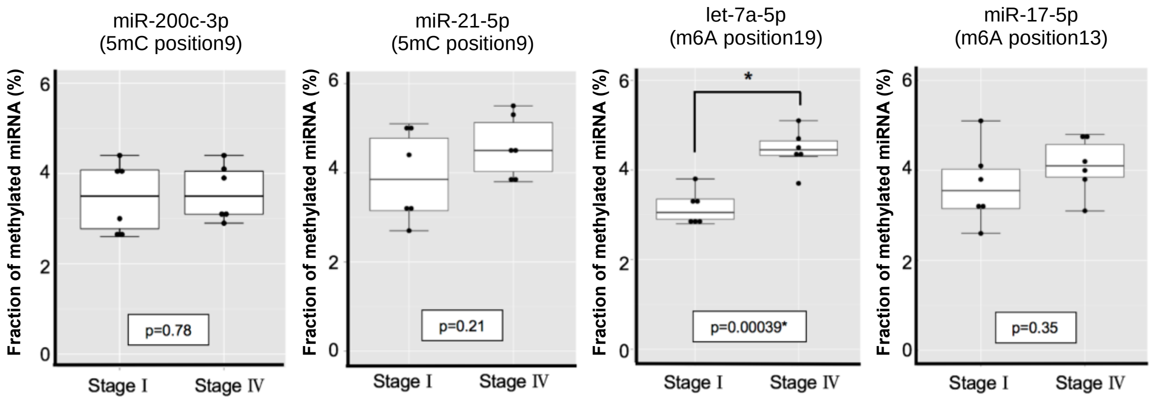

Supplementary Figure 9. Comparison of fraction of methylated miRNAs between stages I and IV colorectal cancer tissues. The fraction of methylated miRNAs at specific positions in each miRNA are classified according to pathological stage. *P < 0.05 (t test). The box range means from the first quartile to the third quartile. The second quartile means the median of the data. The lower limit of the bar was estimated by "the first quartile - 1.5 x interquartile range", and the upper limit of the bar was estimated by "the third quartile + 1.5 x interquartile range". Points means individual data value.

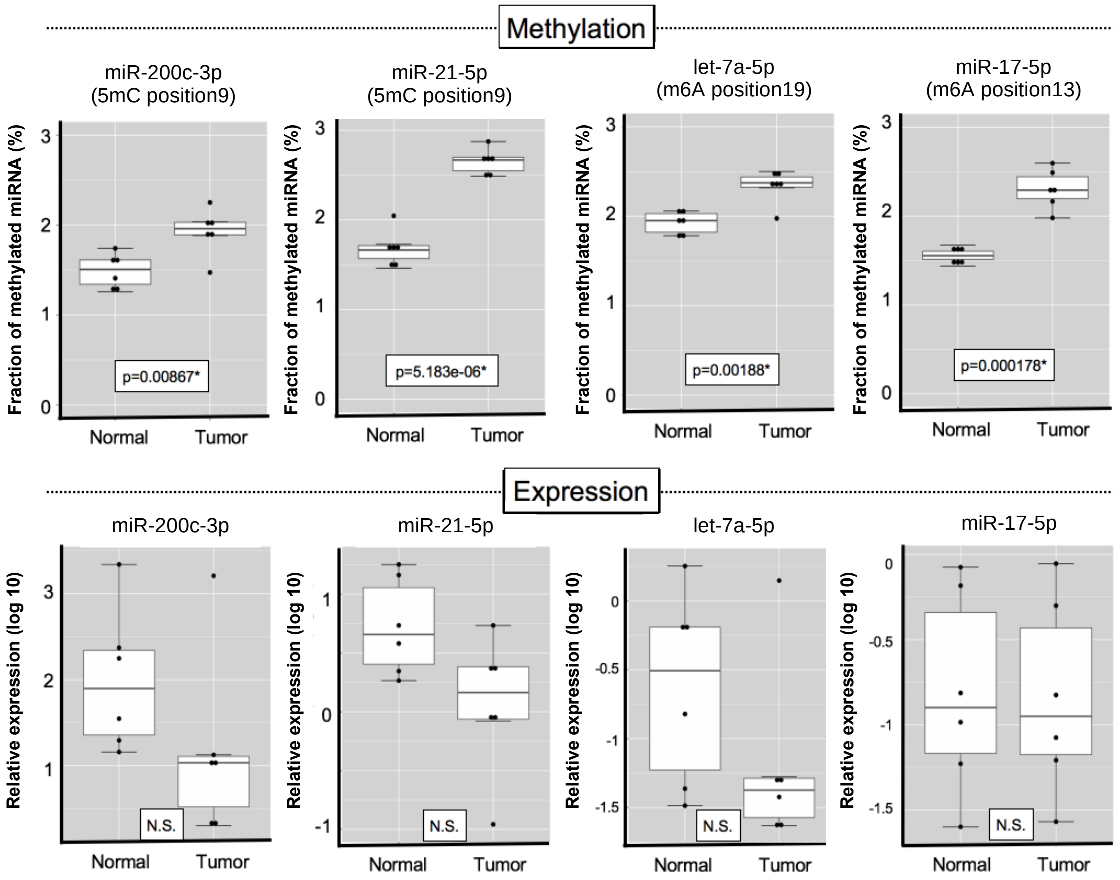

Supplementary Figure 10. Increased miRNA methylation levels in gastric cancer tissues. (Upper panels) Fraction of methylated miRNAs at a specific positions in each miRNA in gastric cancer and paired normal tissues (n = 6). (Lower panels) Expression levels of each miRNA in the same samples determined by qRT-PCR. Detailed clinicopathological information on each patient can be found in Supplementary Table S4. *P < 0.05 (t test). The box range means from the first quartile to the third quartile. The second quartile means the median of the data. The lower limit of the bar was estimated by "the first quartile - 1.5 x interquartile range", and the upper limit of the bar was estimated by "the third quartile + 1.5 x interquartile range". Points means individual data value.

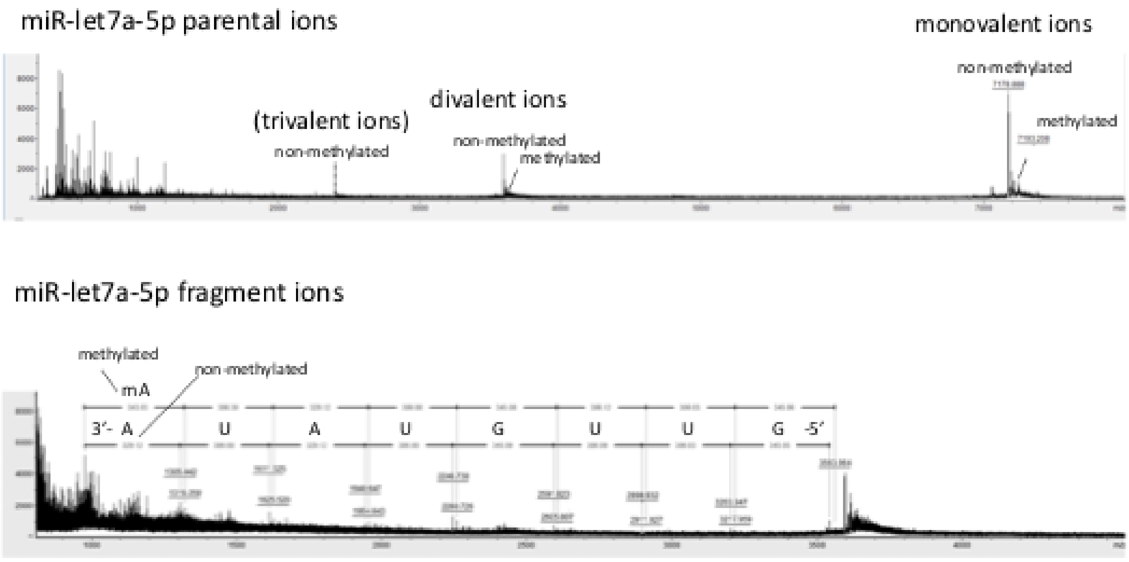

Supplementary Figure 11. Mass spectra of patient serum obtained by MALDI-TOF-MS and MALDI-TOF-MS/MS (let-7a-5p). (Upper panels) Spectrum showing monovalent, divalent, and trivalent ions from methylated and non-methylated let-7a-5p. (Lower panels) MS/MS spectrum of let-7a-5p fragment ions from base 12–19. The adenine at position 19 shows an additional peak (1319–1305 = +14 m/z), indicating methylation (see Supplementary Methods for details).

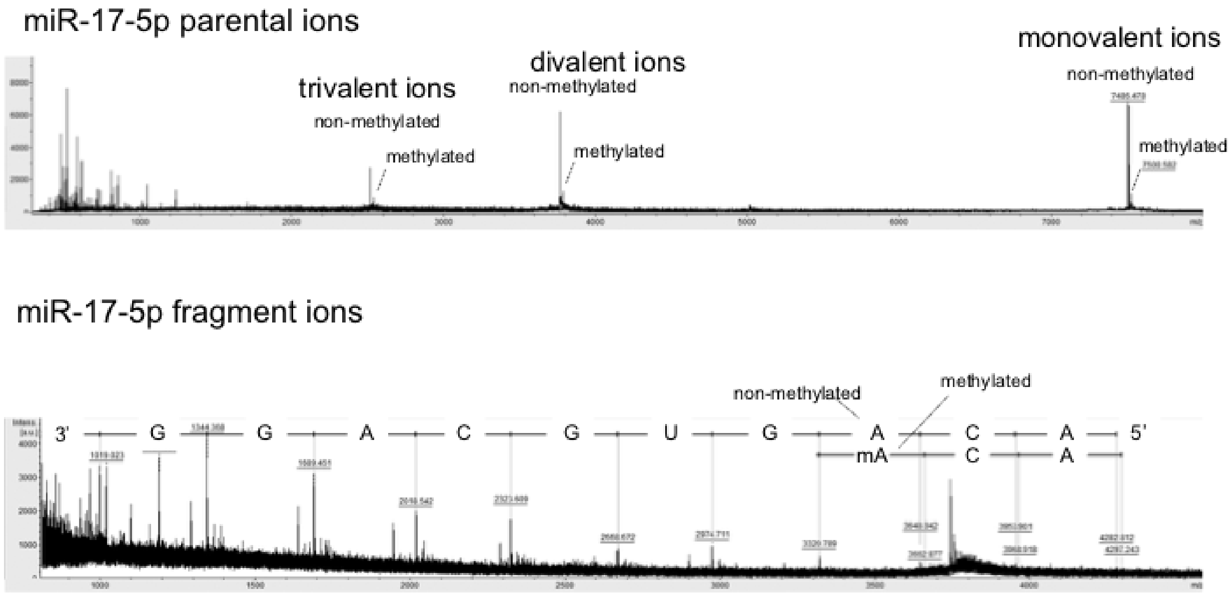

Supplementary Figure 12. Mass spectra of patient serum obtained by MALDI-TOF-MS and MALDI-TOF-MS/MS (miR-17-5p). (Upper panels) Spectrum showing monovalent, divalent, and trivalent ions from methylated and non-methylated let-7a-5p. (Lower panels) MS/MS spectrum of miR-17-5p. Fragment ions from base 11–20 are shown. The adenine at position 13 shows an additional peak (3662–3648 = +14 m/z), indicating methylation (see Supplementary Methods for details).

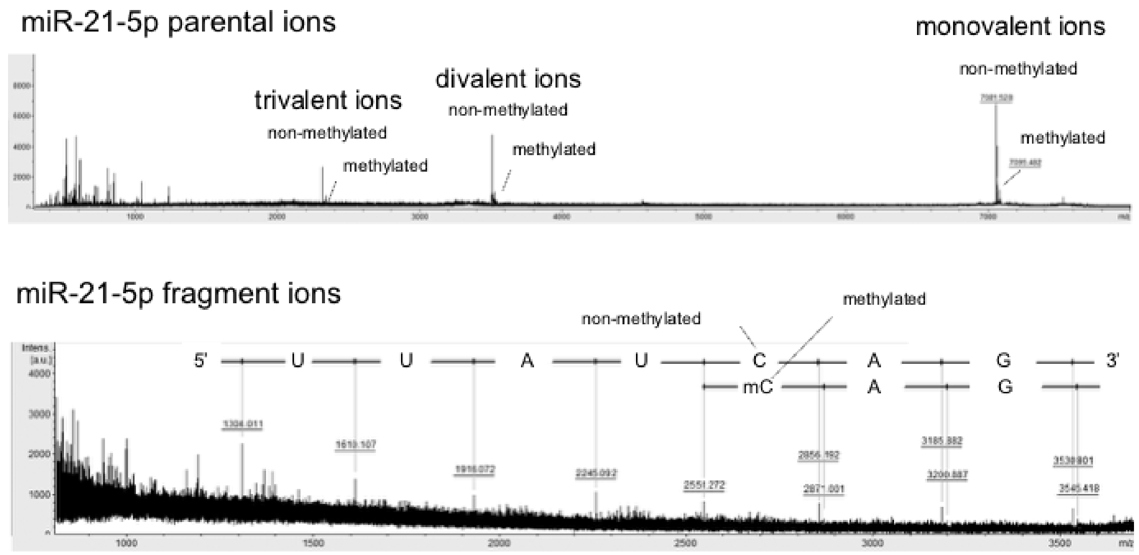

Supplementary Figure 13. Mass spectra of patient serum obtained by MALDI-TOF-MS and MALDI-TOF-MS/MS (miR-21-5p). (Upper panels) Spectrum showing monovalent, divalent, and trivalent ions from methylated and non-methylated miR-21-5p. (Lower panels) MS/MS spectrum of miR-21-5p. Fragment ions from base 5–11 are shown. The cytosine at position 9 showed an additional peak (2856–2870 = +14 m/z), indicating methylation (see Supplementary Methods for details).

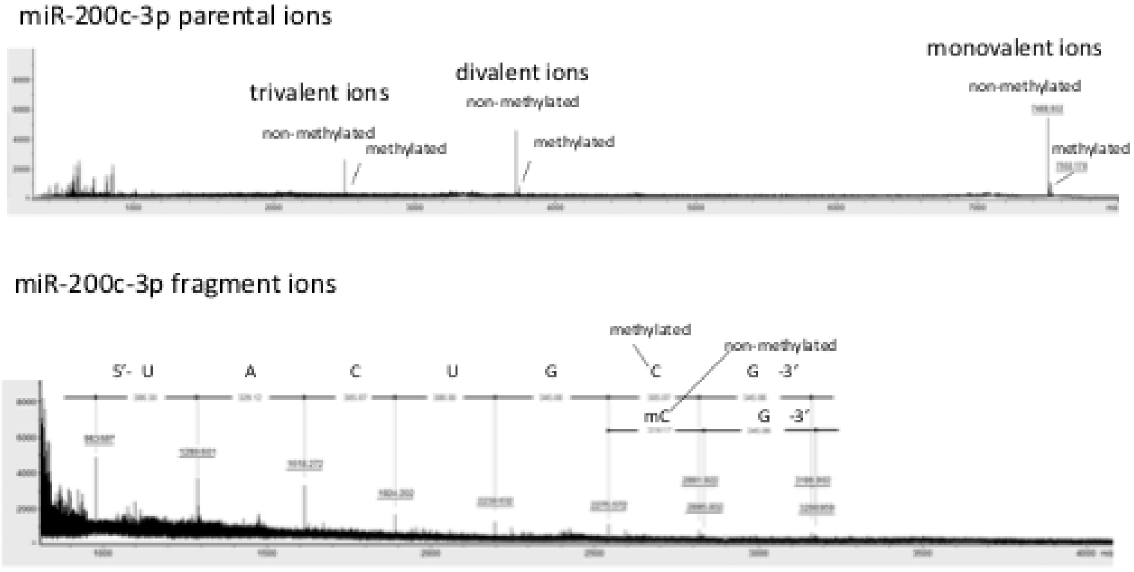

Supplementary Figure 14. Mass spectra of pancreatic cancer tissue obtained by MALDI-TOF-MS and MALDI-TOF-MS/MS (miR-200c-3p). (Upper panels) Spectrum showing monovalent, divalent, and trivalent ions from methylated and non-methylated miR-200c-3p. (Lower panels) MS/MS spectrum showing miR-200c-3p fragment ions from base 4–10. The cytosine at position 9 showed an additional peak (2895–2881 = +14 m/z), indicating methylation (see Supplementary Methods for details).

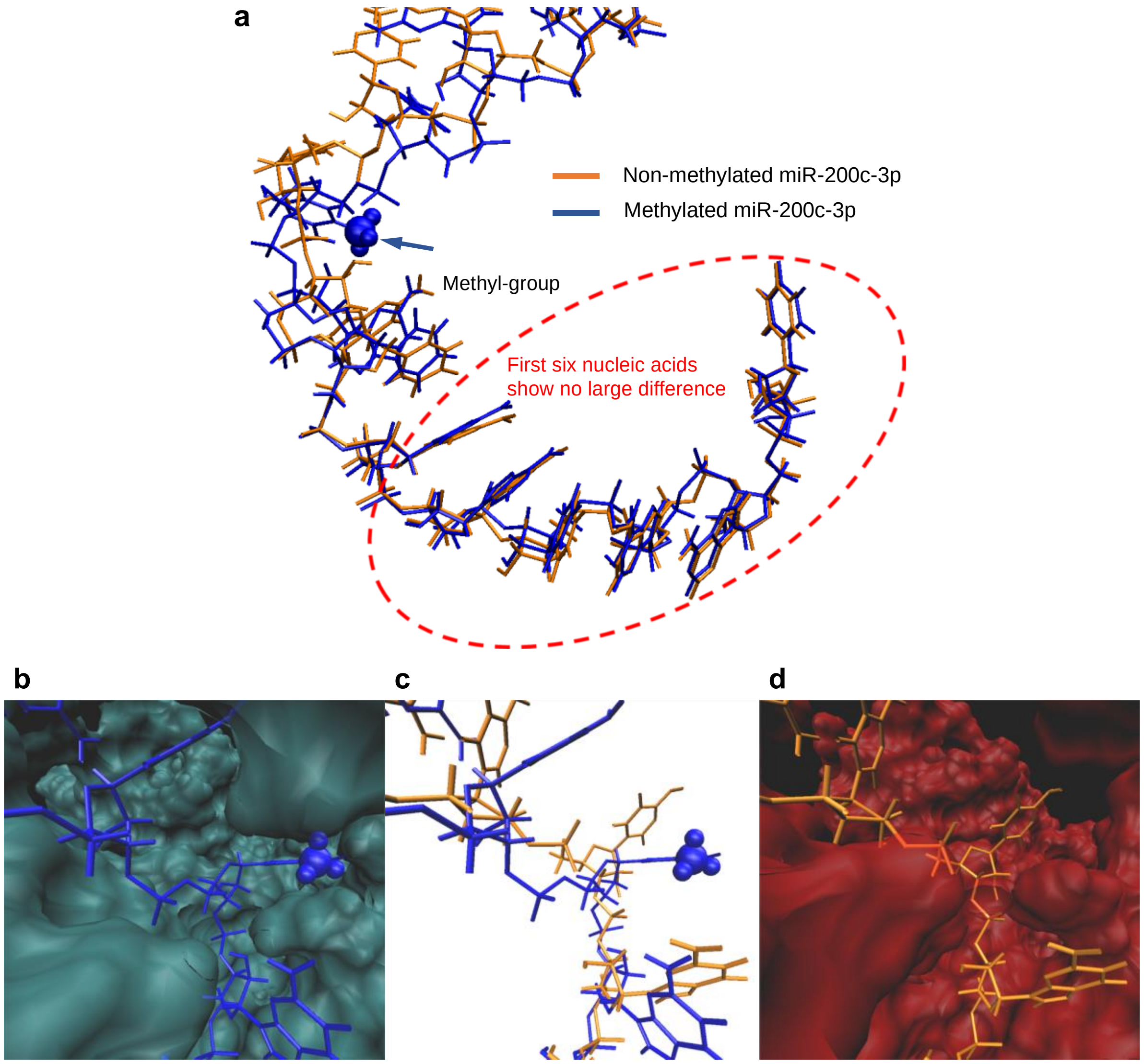

Supplementary Figure 15. Prediction of miR-200c-3p binding to AGO2 protein by a molecular mechanics approach. (a) Superposition of each miRNA is shown for stabilized conformations of each complex, as estimated by energy minimization. Orange and blue bonds represent means for miR-200c-3p and for the methylated body, respectively. There was no significant difference in first six bases; however, a change in the binding interaction was observed around the methyl group. (b) Reduced space around the methyl group due to enhanced van der Waals interactions between the methyl group and AGO2 protein. (c) Altered orientation depending on the presence of a methyl group. (d) Complex of non-methylated miR-200c-3p has more free space than the methylated form.

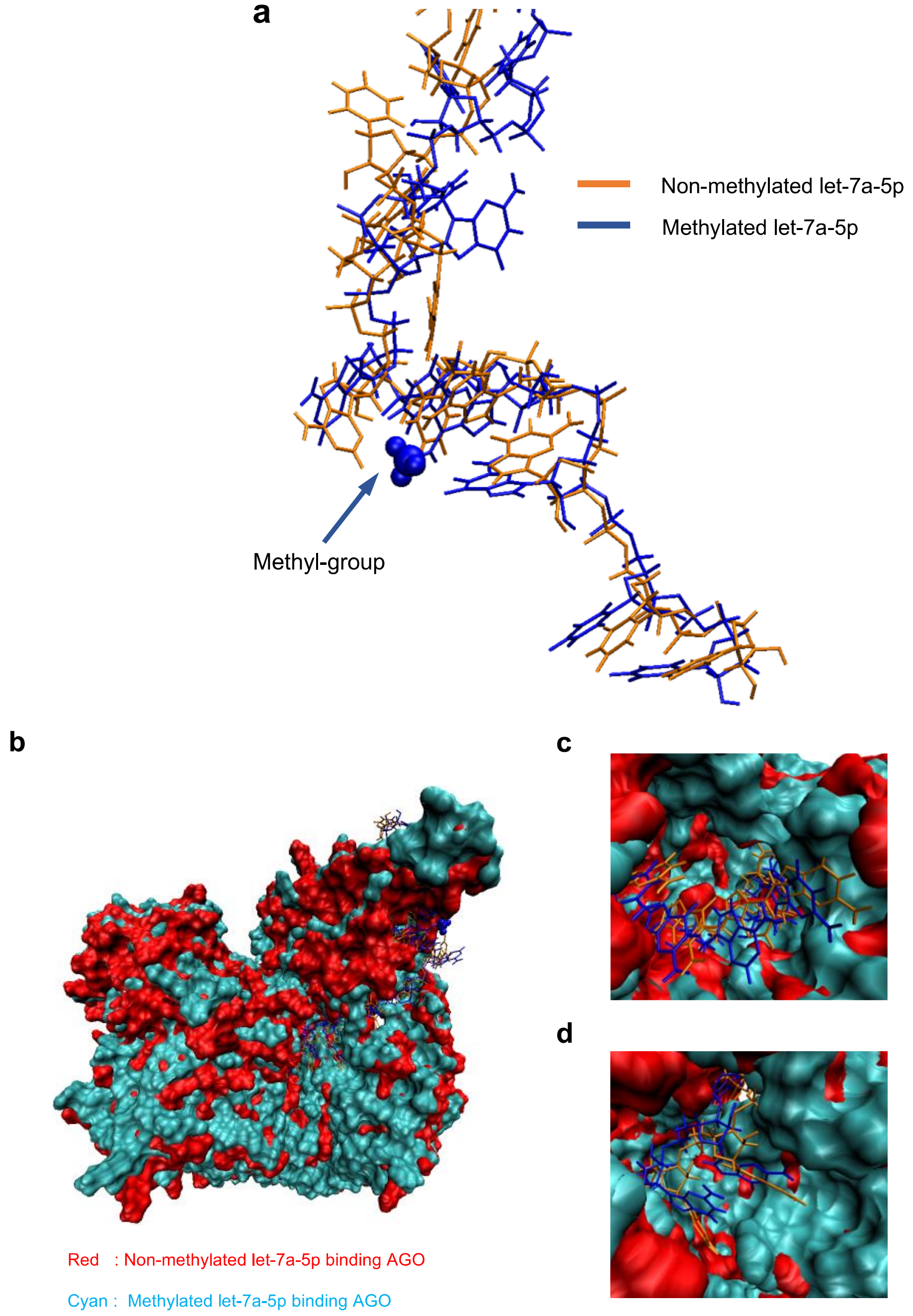

Supplementary Figure 16. Prediction of let-7a-5p binding to AGO2 protein by a molecular mechanics approach. (a) Superposition of each miRNA is shown for stabilized conformations of each complex, as estimated by energy minimization. Orange and blue bonds represent means for let-7a-5p and for the methylated body, respectively. Although the conformations of the backbones were somewhat similar, the orientation of each base differed significantly between non-methylated and methylated forms. (b–d) m6A leads to a structural change in the whole complex (b) and a difference in the space size of the RNA recognition site (c, d).

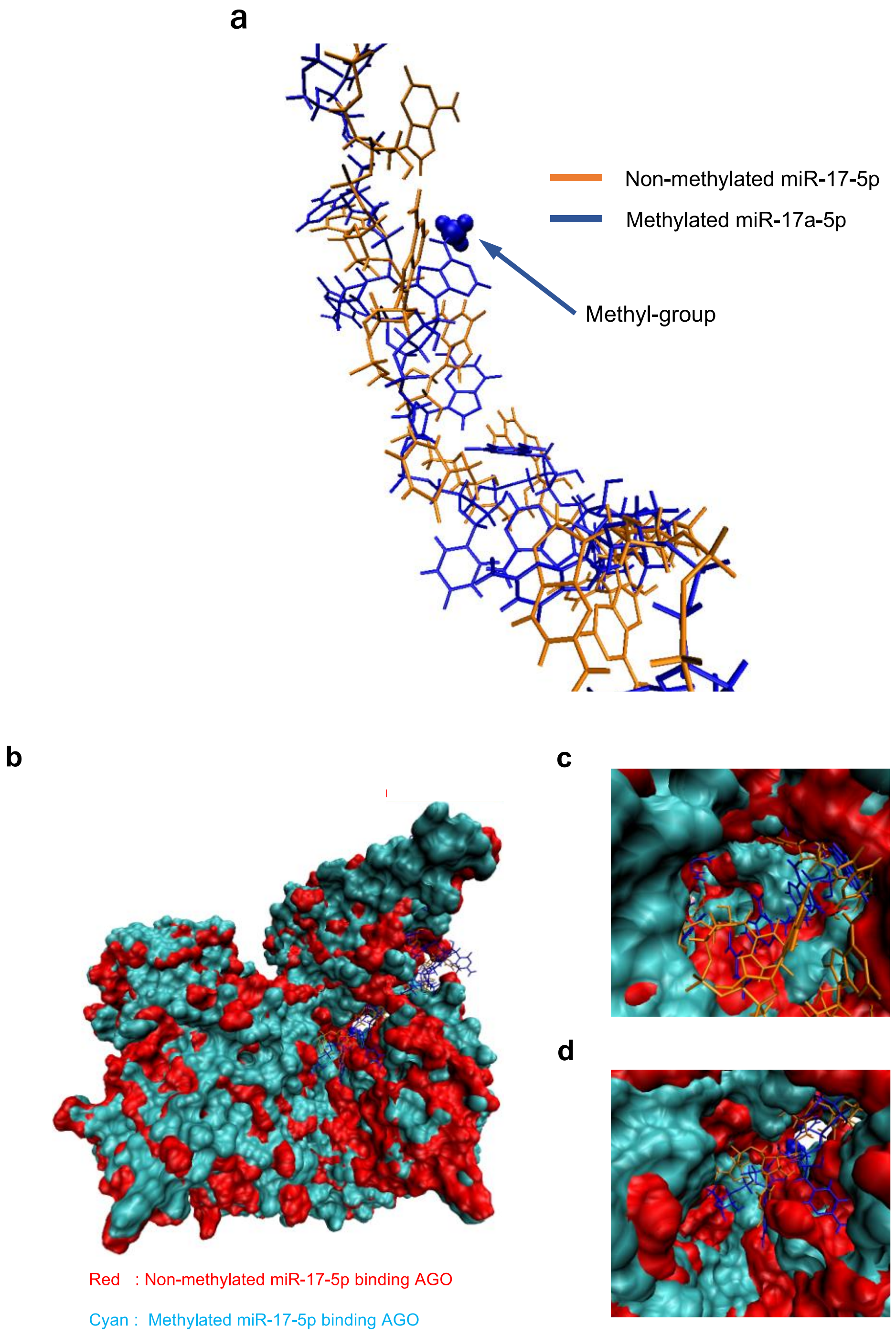

Supplementary Figure 17. Prediction of miR-17-5p binding to AGO2 protein by a molecular mechanics approach. (a) Superposition of each miRNA is shown for stabilized conformations of each complex, as estimated by energy minimization. Orange and blue bonds represent means for miR-17-5p and for the methylated body, respectively. The orientation of the backbone and each base differed significantly between non-methylated and methylated forms. (b–d) As is the case of miR-17-5p, m6A resulted in a structural change in the whole complex (b) as well as a difference in the space size of the RNA recognition site (c, d).

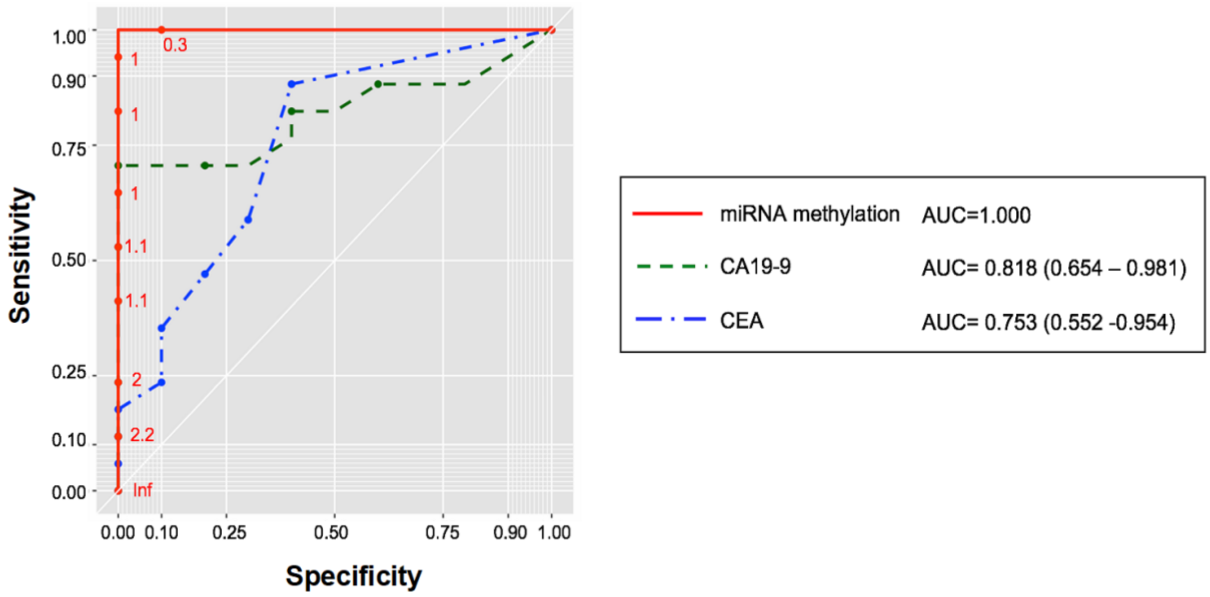

Supplementary Figure 18. Receiver operating characteristic (ROC) curves for miR-17-5p methylation level, CA19-9, and CEA. Numbers beside the curve indicate cut-off values. Area under the ROC curve (AUC) is shown with legends in the box to the right.

Supplementary Table 1. List of miRNAs investigated for methylated level by RNA sequencing following methylated RNA immunoprecipitation

miR	IP average
hsa-let7a	2369110.75
hsa-miR-21	270212.50
hsa-miR-100	136722.00
hsa-miR-222	125231.00
hsa-miR-92a	98972.50
hsa-miR-10a	87338.75
hsa-miR-99b	83181.75
hsa-miR-30d	78856.50
hsa-miR-26a	67485.50
hsa-miR-320a	53015.50
hsa-miR-148a	28775.00
hsa-miR-125a	25089.75
hsa-miR-423	24038.75
hsa-miR-182	23166.50
hsa-miR-7641	22926.75
hsa-miR-378a	17505.00
hsa-miR-1307	16547.75
hsa-miR-221	15846.75
hsa-miR-183	14621.25
hsa-miR-25	13353.00
hsa-miR-24	12976.25
hsa-miR-30a	11938.00
hsa-miR-128	10923.25
hsa-miR-941	10571.50
hsa-miR-1246	9965.00
hsa-miR-92b	8120.75
hsa-miR-122	7972.50
hsa-miR-5100	7827.50
hsa-miR-106b	7492.25
hsa-miR-181a	7321.75
hsa-miR-27b	7140.75
hsa-miR-29a	7051.25

miR	IP average
hsa-miR-224	6763.50
hsa-miR-191	6311.00
hsa-miR-146b	5709.00
hsa-miR-27a	5086.50
hsa-miR-3182	5045.50
hsa-miR-532	4561.00
hsa-miR-3184	4464.75
hsa-miR-30c	4148.75
hsa-miR-181b	4136.75
hsa-miR-744	3675.25
hsa-miR-7706	3366.75
hsa-miR-148b	3097.00
hsa-miR-629	2873.00
hsa-miR-103b	2707.50
hsa-miR-103a	2664.50
hsa-miR-98	2661.25
hsa-miR-23a	2335.25
hsa-miR-425	2225.50
hsa-miR-192	2207.75
hsa-miR-22	2177.50
hsa-miR-3615	1955.75
hsa-miR-5701	1907.75
hsa-miR-155	1817.00
hsa-miR-149	1250.00
hsa-miR-7704	1156.00
hsa-miR-1180	1089.50
hsa-miR-1275	965.00
hsa-miR-769	955.25
hsa-miR-1273g	920.75
hsa-miR-484	914.75
hsa-miR-17	875.50

The miRNAs we analyzed in the present study are highlighted in yellow.

Supplementary Table 2. Dynamic range of detection of methylated miRNAs

Spiked in (%)	Detected(%)	S.D.
0.1000	0.0937	0.0013
1.0000	0.9881	0.0188
5.0000	4.8934	0.0944
10.0000	9.7870	0.3184

S.D., standerd deviation

Supplementary Table 3. Clinicopathological characteristics of colorectal cancer patients

Sample number	Location	Tumor depth	Lymph node metastasis	Distant metastasis	Pathological stage*
No.1	Rectum	T2	N0	-	I
No.2	Rectum	T2	N0	-	I
No.3	Colon	T2	N0	-	I
No.4	Rectum	T2	N0	-	I
No.5	Colon	T1	N0	-	I
No.6	Colon	T2	N0	-	I
No.7	Rectum	T3	N3	Liver metastasis	IV
No.8	Colon	T3	N2	Liver metastasis	IV
No.9	Rectum	T3	N1	Liver metastasis	IV
No.10	Rectum	T3	NA	Liver metastasis	IV
No.11	Colon	T4a	NA	Liver metastasis	IV
No.12	Rectum	NA	NA	Liver metastasis	IV

*Tumor-node-metastasis (TNM) classification was according to the 7th edition of TNM staging of the Union for International Cancer Control (https://www.nccn.org/professionals/physician_gls/f_guidelines.asp).

NA, not available.

Supplementary Table 4. Clinicopathological characteristics of gastric cancer patients

Sample number	Histology	Tumor depth	Lymph node metastasis	Distant metastasis	Pathological stage*
No.1	Tubular adenocarcinoma	T3	N1	0	ⅡB
No.2	Tubular adenocarcinoma	T3	N2	0	ⅢA
No.3	Tubular adenocarcinoma	T3	N3a	0	ⅢB
No.4	Signet-ring cell carcinoma	T4a	N3a	0	ⅢC
No.5	Tubular adenocarcinoma	T4a	N3b	0	ⅢC
No.6	Endocrine carcinoma	T4a	N1	0	ⅢA

*Tumor-node-metastasis (TNM) classification was according to the 7th edition of TNM staging of the Union for International Cancer Control (https://www.nccn.org/professionals/physician_gls/f_guidelines.asp).

Supplementary Table 5. Clinicopathological characteristics of pancreatic cancer patients

Sample No.	Clinical stage before surgery	T	N	M	Pathological stage*
No. 1	ⅠA	Tis	0	0	0
No. 2	ⅠA	1	0	0	ⅠA
No. 3	ⅡA	1	0	0	ⅠA
No. 4	ⅡA	1	0	0	ⅠA
No. 5	ⅡA	1	0	0	ⅠA
No. 6	ⅡA	3	0	0	ⅡA
No. 7	ⅡA	3	0	0	ⅡA
No. 8	ⅡA	3	0	0	ⅡA
No. 9	ⅡA	3	0	0	ⅡA
No. 10	ⅡA	3	0	0	ⅡA
No. 11	ⅡA	3	1	0	ⅡB
No. 12	ⅡA	3	1	0	ⅡB
No. 13	ⅠA	3	1	0	ⅡB
No. 14	ⅡA	3	1	0	ⅡB
No. 15	ⅡB	3	1	0	ⅡB
No. 16	ⅡA	2	1	0	ⅡB
No. 17	ⅠA	3	1	0	ⅡB

* Tumor-node-metastasis (TNM) classification was according to the 7th edition of TNM staging of the Union for International Cancer Control (https://www.nccn.org/professionals/physician_gls/f_guidelines.asp). T, N, and M scores are those of pathological diagnosis.

Tis, carcinoma in situ.
