# Supplementary material for: Distinct methylation levels of mature microRNAs in gastrointestinal cancers
Source: Nat Commun. 2019 Aug 29;10:3888. doi: 10.1038/s41467-019-11826-1 (PMC6715669; doi:10.1038/s41467-019-11826-1)
Supplement: Supplementary file 2 — Reporting Summary [file 41467_2019_11826_MOESM2_ESM.pdf]

nature research

Corresponding author(s): Masaki Mori, Ken Ofusa, Hideshi Ishii

Last updated by author(s): Aug 3, 2019

Reporting Summary

Nature Research wishes to improve the reproducibility of the work that we publish. This form provides structure for consistency and transparency in reporting. For further information on Nature Research policies, see Authors & Referees and the Editorial Policy Checklist.

Statistics

For all statistical analyses, confirm that the following items are present in the figure legend, table legend, main text, or Methods section.

n/a	Confirmed	
☐	☒	The exact sample size (n) for each experimental group/condition, given as a discrete number and unit of measurement
☐	☒	A statement on whether measurements were taken from distinct samples or whether the same sample was measured repeatedly
☐	☒	The statistical test(s) used AND whether they are one- or two-sided<br>*Only common tests should be described solely by name; describe more complex techniques in the Methods section.*
☐	☒	A description of all covariates tested
☐	☒	A description of any assumptions or corrections, such as tests of normality and adjustment for multiple comparisons
☐	☒	A full description of the statistical parameters including central tendency (e.g. means) or other basic estimates (e.g. regression coefficient) AND variation (e.g. standard deviation) or associated estimates of uncertainty (e.g. confidence intervals)
☐	☒	For null hypothesis testing, the test statistic (e.g. F, t, r) with confidence intervals, effect sizes, degrees of freedom and P value noted<br>*Give P values as exact values whenever suitable.*
☐	☒	For Bayesian analysis, information on the choice of priors and Markov chain Monte Carlo settings
☐	☒	For hierarchical and complex designs, identification of the appropriate level for tests and full reporting of outcomes
☐	☒	Estimates of effect sizes (e.g. Cohen's d, Pearson's r), indicating how they were calculated

Our web collection on statistics for biologists contains articles on many of the points above.

Software and code

Policy information about availability of computer code

Data collection	We do not use the special software for data collection.
Data analysis	We do not use the special program to analyses our data.

For manuscripts utilizing custom algorithms or software that are central to the research but not yet described in published literature, software must be made available to editors/reviewers. We strongly encourage code deposition in a community repository (e.g. GitHub). See the Nature Research guidelines for submitting code & software for further information.

Data

Policy information about availability of data

All manuscripts must include a data availability statement. This statement should provide the following information, where applicable:

- Accession codes, unique identifiers, or web links for publicly available datasets
- A list of figures that have associated raw data
- A description of any restrictions on data availability

The data that support the findings of this study are available from the corresponding authors upon request. Sequence data for Figure 1d have been deposited in the NCBI GEO data set with accession code GSE119014 (https://www.ncbi.nlm.nih.gov/geo/query/acc.cgi?acc=GSE119014); Microarray data for Supplementary Figure 4 have been deposited in the NCBI GEO data set with accession code GSE134794 (https://www.ncbi.nlm.nih.gov/geo/query/acc.cgi?acc=GSE134794). Public datasets used in this study can be found in GEO under the accession numbers: GDS4103 (https://www.ncbi.nlm.nih.gov/geo/query/acc.cgi?acc=GSE15471), and GDS4382 (https://www.ncbi.nlm.nih.gov/geo/query/acc.cgi?acc=GSE32323). X-ray structure used in this study can be found in PDB ID: 4OLB (http://www.rcsb.org/structure/4OLB) and 4W5N2 (http://www.rcsb.org/structure/4W5N).

Field-specific reporting

Please select the one below that is the best fit for your research. If you are not sure, read the appropriate sections before making your selection.

☒ Life sciences ☐ Behavioural & social sciences ☐ Ecological, evolutionary & environmental sciences

For a reference copy of the document with all sections, see nature.com/documents/nr-reporting-summary-flat.pdf

Life sciences study design

All studies must disclose on these points even when the disclosure is negative.

Sample size	No sample-size calculation was performed. We used the available samples stored at Osaka University Hospital.
Data exclusions	No data were excluded from the analysis.
Replication	All samples were analyzed more than 3 times. We confirmed that all data have replication.
Randomization	Colorectal and gastric cancer and paired normal tissue samples (located > 5 cm from the malignant region) were obtained during surgery. All patients underwent resection of the primary tumor at Osaka University Hospital (Osaka, Japan) and affiliated hospitals. Blood samples from pancreatic cancer patients were collected before surgery. Healthy controls had no visible lesions by diagnostic imaging and no abnormal findings in the blood examination. Informed consent was obtained from all participants.
Blinding	M.K., A.Y., and K.O. performed mass spectrometry analysis in the situation where we do not know clinical information

Reporting for specific materials, systems and methods

We require information from authors about some types of materials, experimental systems and methods used in many studies. Here, indicate whether each material, system or method listed is relevant to your study. If you are not sure if a list item applies to your research, read the appropriate section before selecting a response.

Materials & experimental systems

n/a	Involved in the study	
☐	☒	Antibodies
☐	☒	Eukaryotic cell lines
☒	☐	Palaeontology
☒	☐	Animals and other organisms
☐	☒	Human research participants
☒	☐	Clinical data

Methods

n/a	Involved in the study	
☒	☐	ChIP-seq
☒	☐	Flow cytometry
☒	☐	MRI-based neuroimaging

Antibodies

Antibodies used	anti-m6A antibody (cat. 202 003, Synaptic Systems, Goettingen, Germany)
Validation	Validation statements were noted on manufacture’s web site. (https://www.sysy.com/products/m6a/facts-202003.php)

Eukaryotic cell lines

Policy information about cell lines

Cell line source(s)	BxPC3, Panc10.5, PSN1, Capan2, HCT116, and HCT116EX5
Authentication	BxPC3, Panc10.5, PSN1, and Capan2 human pancreatic cancer cell lines were obtained from American Type Culture Collection (Manassas, VA, USA). HCT116EX5 cells were a gift from Bert Vogelstein (Johns Hopkins University, Baltimore, MS, USA).
Mycoplasma contamination	Mycoplasma testing was performed using the MycoAlert Mycoplasma Detection Kit (Lonza, Allendale, NJ). Mycoplasma testing confirmed negative results.
Commonly misidentified lines (See ICLAC register)	In this study, no commonly misidentified cell lines were used.

Human research participants

Policy information about studies involving human research participants

Population characteristics	Colorectal and gastric cancer and paired normal tissue samples (located > 5 cm from the malignant region) were obtained during surgery. All patients underwent resection of the primary tumor at Osaka University Hospital (Osaka, Japan) and affiliated hospitals.
Recruitment	Blood samples from pancreatic cancer patients were collected before and after surgery. Healthy controls had no visible lesions by diagnostic imaging and no abnormal findings in the blood examination.
Ethics oversight	We have complied with all relevant ethical regulations for work with human participants. The study protocols were approved by the ethics committee of Osaka University (approval nos. 663, 15222-2, and 08226-5).

Note that full information on the approval of the study protocol must also be provided in the manuscript.
